# Evidence of motor system reorganization in complex regional pain syndrome type 1: A case report

**DOI:** 10.1080/24740527.2017.1422116

**Published:** 2018-01-30

**Authors:** Marie-Philippe Harvey, Samuel Maher-Bussières, Elysa Emery, Marylie Martel, Francis Houde, Yannick Tousignant-Laflamme, Guillaume Léonard

**Affiliations:** a Faculty of Medicine and Health Sciences, University of Sherbrooke, Sherbrooke, Québec, Canada; b Research Centre on Aging, University Institute of Geriatrics of Sherbrooke, Sherbrooke, Québec, Canada; c Clinical Research Centre Étienne–Le Bel-CHUS, Sherbrooke, Québec, Canada; d School of Rehabilitation, Faculty of Medicine and Health Sciences, University of Sherbrooke, Sherbrooke, Québec, Canada

**Keywords:** complex regional pain syndrome, transcranial magnetic stimulation, graded motor imagery

## Abstract

**Background:**

Central nervous system reorganization, particularly in networks devoted to somatosensation, is thought to be a significant feature of complex regional pain syndrome (CRPS).

**Aims:**

In the present case report, we evaluated the corticomotor system of a woman suffering from CRPS, as she started and completed her rehabilitation, in order to explore whether CRPS could also be linked to changes in motor networks.

**Methods:**

The patient, a 58-year-old woman, was diagnosed with right-hand CRPS. Transcranial magnetic stimulation measures, reflecting the strength of the corticospinal projections, were evaluated before, during, and after an 8-week graded motor imagery (GMI) program.

**Results:**

Before treatment, the patient reported significant pain and disability, and the strength of the corticospinal projections of the first dorsal interosseous of the affected hand was reduced compared to the healthy, unaffected hand. Pain and disability decreased as the patient completed the GMI program. These changes were paralleled by an increase in the strength of the corticospinal projections.

**Conclusions:**

These observations suggest that corticomotor changes can be observed in individuals suffering from CRPS and that some of the clinical manifestations observed in these patients (e.g., pain, disability) could possibly be linked to these neurophysiological changes.

## RÉSUMÉ

**Contexte:** La réorganisation du système nerveux central des réseaux cérébraux, en particulier ceux liés à la somatosensation, est connue comme une caractéristique importante du syndrome douloureux régional complexe (SDRC).

**But:** Dans la présente étude de cas, nous avons évalué le système corticomoteur d’une femme souffrant du SDRC, du début à la fin de sa réadaptation, afin de déterminer si le SDRC pouvait aussi être lié à des changements dans les réseaux moteurs.

**Méthode:** La patiente, une femme âgée de 58 ans, avait reçu un diagnostic de SDRC au niveau de la main droite. Des mesures de stimulation magnétique transcrânienne reflétant la force des projections corticospinales ont été évaluées avant, pendant et après un programme d’imagerie motrice progressive (IMP) d’une durée de huit semaines.

**Résultats:** Avant le traitement, la patiente rapportait douleur et une incapacité importantes, et la force de ses projections corticospinales du premier interosseux dorsal de la main affectée, comparativement à la main saine, non affectée était réduite. La douleur et l’incapacité ont diminué pendant le programme d’IMP. Parallèllement à ces changements, une augmentation de la force des projections corticospinales a été observée.

**Conclusions:** Ces observations suggèrent que des changements corticomoteurs peuvent être observés chez des individus souffrant de SDRC, et que certaines manifestations cliniques observées chez ces patients (ex.: la douleur, l’incapacité) pourraient être liées à ces changements neurophysiologiques.

## Introduction

Complex regional pain syndrome (CRPS) is an amplified pain syndrome usually occurring after physical trauma with (type II) or without (type I) demonstrable nerve damage.^1^ The condition is characterized by sensorimotor disturbances leading to a loss of motor function and movement disorders that are associated with a spread of sensory symptoms in the extremities, such as intense pain, autonomic dysregulation, paresthesia, and numbness.^2–4^ Although pain is the primary symptom, motor manifestations, such as muscle weakness and dystonia, are frequently encountered in patients with CRPS.^5,6^

**Figure 1.**
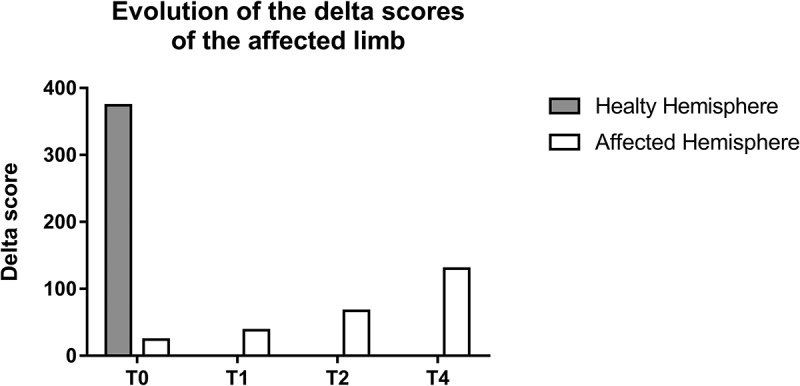
Evolution of the delta score of the affected limb. The delta score was used to depict the strength of the corticospinal projections, with a higher delta score representing a higher strength of corticospinal projections (delta score = mean MEP amplitude at 130% – mean MEP amplitude at 110%). T0 corresponds to the score obtained at the initial visit (prior to GMI treatment), T1 to the score obtained after 2 weeks (stage 1 GMI), T2 to the score obtained after 4 weeks (stage 2 GMI) and T4 to the score obtained after 8 weeks (stage 4 GMI).

The underlying pathophysiological mechanism of CRPS has not been identified, although many authors argue that central nervous system (CNS) reorganization may play a considerable role.^7,8^ Patients with CRPS show cortical reorganization of the primary (SI) and secondary somatosensory cortex (SII) contralateral to the affected limb.^8^ Restoration of cortical map size in contralateral SI/SII after CRPS treatments has been associated with reduced pain symptoms, suggesting that cortical reorganization is a key pathological feature of CRPS.^7^

Some studies suggest that these cortical changes are not restricted to somatosensory brain areas but can also be observed in adjacent motor regions.^9–15^ In one of these studies, Krause and colleagues used transcranial magnetic stimulation (TMS) to map the motor cortex of individuals suffering from CRPS type 1.^14^ They observed a significant asymmetry between the affected and unaffected hemispheres, with the corticomotor representation corresponding to the unaffected hand being significantly larger than that of the affected hand. Other TMS studies also revealed that intracortical inhibition (a measure reflecting the activity of intracortical motor networks) is decreased in patients with CRPS.^9,13^ In their report, Schwenkreis and colleagues noted that this reduction in intracortical inhibition was linked to pain severity.^9^ At this point, however, it remains difficult to determine whether these cerebral changes are a cause or a consequence of the pain syndrome. To our knowledge, no studies followed these changes longitudinally and looked at how corticomotor alterations evolved with time as the condition improved.

Mirror therapy was introduced as a treatment for CRPS in the early 2000s.^16,17^ In order to be coherent with the sequential activation of the motor cortex, Moseley suggested that mirror therapy should be preceded by hand laterality recognition exercises.^17^ This addition by Moseley led to the graded motor imagery (GMI) program. GMI, consisting of laterality recognition, imagined movements, and mirror movements, proved successful to reduce pain and disability in patients with CRPS.^7,17^ Interestingly, several elements of the GMI program (e.g., observation, imagery) have been shown to activate the motor cortices and their descending projections,^18,19^ highlighting the need to better understand the role played by the motor system in CRPS.

## Materials and methods

### History

The patient was a 58-year-old woman diagnosed with CRPS-I, according to Budapest criteria, 10 weeks after she suffered a radial (right) fracture due to a fall. No surgery was needed for the fracture, and the limb was immobilized with a splint and traction for 6 weeks. After removal of the splint, the patient received conventional physical and occupational therapy for 8 weeks with the aim to improve the mobility and pain of the affected limb. Upon initial assessment, 9 weeks after removal of the splint, the patient showed hyperalgesia, paresthesia, minor edema, and increased sweating. Her affected hand was also warmer, and there was skin color asymmetry. Compared to the unaffected hand, the patient showed reduced strength and decreased range of motion. No dystonia or any other movement disorder was noted. The patient was taking 50 mg of pregabalin in the morning and 75 mg at night. No change in medication occurred during GMI treatments. The patient was asked to refrain from consuming caffeine for 6 h before testing and from using tobacco products for 2 h before testing. The patient reported not having consumed any alcohol for the last 12 months before starting the treatment.

The study was approved by the local institutional ethics committee and written informed consent was obtained from the patient. All procedures performed in studies were in accordance with the ethical standards of the local institutional research committee and with the 1964 Helsinki Declaration and its later amendments or comparable ethical standards.

### Modified graded motor imagery treatments

Modified GMI therapy included four phases: (1) recognition of laterality—the patient identifies right or left hands displayed on a screen; (2) mental imagery—the patient imagines taking the positions shown on the screen with the affected limb; (3) mirror therapy—the patient moves the unaffected hand in the position of hands presented on the screen, while looking at the reflection of the unaffected hand in the mirror; and (4) mirror box therapy—the patient moves both hands in the position of the hands presented on the screen, while the affected hand is covered by a box and the patient watches the reflection of the unaffected hand. Treatment lasted 8 weeks (2 weeks for each of the four GMI phases). The procedure was shown to the patient at the beginning of each phase. The patient performed the exercises at home for 10 min 3 times/day, 6 days/week.

### Clinical outcome measures

Pain was evaluated with the short form of the McGill Pain Questionnaire (SF-MPQ). Perception of upper limb function was assessed with the Disabilities of the Arm, Shoulder and Hand questionnaire (DASH). Maximal grip strength was evaluated with a Martin vigorimeter. These evaluations were performed before and after the completion of the 8 weeks of GMI treatment.

### Corticospinal measures

Corticospinal measures of the affected hand were assessed with TMS before, during (after phases 1 and 2), and after GMI. Motor evoked potentials (MEPs) were elicited in the first dorsal interosseous (FDI) using a 70-mm figure-of-eight coil connected to a Magstim 200 TMS device (Magstim Compagny Ltd., Whitland, UK) and recorded with surface electrodes. The signal was amplified and filtered (bandwidth, 200 Hz to 2 kHz) with a CED 1902 amplifier (Cambridge Electronic Design Limited, Cambridge, UK) and digitized at a sampling rate of 10 kHz with a Power 1401 mk II interface and Spike 2 software (version 7.10; Cambridge Electronic Design Limited). The optimal location for eliciting MEPs in the FDI was found (hotspot). This site was then marked on the swim cap worn by the patient to ensure consistent coil positioning. At this point, stimulations of varying intensities were sent to determine the resting motor threshold (rMT), defined as the minimal intensity of stimulation capable of eliciting MEPs of at least 50% V in 50% of the trials with the FDI at rest. Then, with the subject at rest, eight MEPs were recorded at 110% and 130% of the rMT, with at least 5%s between stimulations. The relationship between stimulus intensity and MEP amplitude (a measure believed to reflect the strength of the corticospinal projections) was assessed by calculating delta scores.^20^ The delta score was used to depict the input–output recruitment curves (a higher delta score represents a higher strength of corticospinal projections).^20^ Calculation of the delta score was done between the mean MEP amplitude obtained at 130% of the rMT and the mean MEP amplitude obtained at 110% of the rMT (delta score = mean MEP amplitude at 130% − mean MEP amplitude at 110%; see Lefaucheur et al.^21^ for a similar approach).

## Results

### Clinical outcomes

Pain intensity, measured with the SF-MPQ 10 cm visual analog scale (VAS), was 4.7 for the initial visit and reduced to 2.5 at the end of the GMI program (Table 1). The pain rating index (PRI) of the SF-MPQ decreased from 11/45 at the initial visit to 3/45 after GMI. Prior to the GMI program, the patient perceived 56% of disability in her affected limb according to the DASH (Table 1). At the end of the GMI program, the evaluation of disability was reduced to 28%. Grip force was 5.4 kg for the healthy hand compared to 0.0 kg for the affected hand upon initial visit (Table 1). Maximal grip strength of the affected hand slightly increased to 0.4 kg after GMI treatment. No changes were noted between the initial and last visit for edema and for other sudomotor/vasomotor manifestations.

**Table 1.** Clinical outcomes.

	Before GMI	After GMI	Difference(before − after)	MCID
SF-MPQ VAS	4.7	2.5	2.2	2/10
SF-MPQ PRI	11	3	8	5/45
DASH (%)	55.8	27.5	28.3	10.83
Grip force (kg)	0	0.4	0.4	5

GMI = graded motor imagery; MCID = minimal clinically important difference; SF-MPQ = short form of the McGill Pain Questionnaire; VAS = visual analogue scale; PRI = pain rating index; DASH = Disabilities of the Arm, Shoulder and Hand questionnaire.

### Corticospinal measures

Before the GMI treatment, the delta score (reflecting the strength of the corticospinal tract) of the affected hand was substantially reduced compared to that of the healthy hand. As can be seen from Figure 1, a progressive increase in the delta score of the affected hand was observed during treatment. The initial delta score continued to increase until the end of the GMI program but was still considerably lower than the delta score for the healthy hand.

## Discussion

Over the past few years, growing evidence suggests that CNS reorganization is a pathological hallmark of CRPS.^22^ To date, these changes were mainly documented in the somatosensory system, namely, the primary and secondary somatosensory cortices.^8^ In this case report, we provide preliminary evidence that motor systems can also be affected in CRPS patients. More specifically, our data suggest that CRPS might be linked to a decrease in the strength of the corticospinal tract, as depicted by the delta score measured with TMS. Interestingly, the delta score increased following completion of a GMI program and paralleled the clinical improvements noted after treatment (increased grip strength, decreased disability, and pain).

These observations are somewhat reminiscent of the results of Pleger et al., who showed that the initiation of a sensorimotor treatment program in a group of patients suffering from CRPS type I decreased pain, improved tactile discrimination, and normalized the pathological changes observed in SI/SII.^8^ The results of this case report extend the observations of Pleger and colleagues by showing that CRPS can affect both the sensory and motor systems. The presence of changes in both the sensory and motor systems is not surprising considering the extensive amount of reciprocal connections observed between M1 and S1.^23^ The results of this case report also concur with previous studies that reported the involvement of the motor system in CRPS.^9,13–15^

A substantial decrease in the strength of the corticospinal tract of the affected hand was still observed at the end the GMI treatment, compared to the healthy hand, suggesting that improvements in pain, disability, and cortical representation could still be achieved with longer-term treatments. A follow-up until complete remission (no pain) would have been interesting to assess longer-term effects.

Although we like to suggest that the changes noted in TMS-evoked MEP amplitudes are signs reflecting a normalization of the corticomotor system, great care should be taken to avoid oversimplification and potential misinterpretation. Indeed, many factors are known to affect corticospinal excitability, and changes in TMS measures over time can be attributable to various other elements and thus cannot automatically be interpreted as indicative of a normalization of the motor system. It should also be said that the current observations do not allow us to come to any conclusions about the presence of causal relationships. For example, it is possible that the changes in pain noted by the patient encouraged her to use her affected hand more frequently, a situation that could, on its own, foster neuroplastic changes in the motor system.^24,25^

The GMI program reduced pain intensity (VAS of the SF-MPQ) and the qualitative descriptors of pain (PRI of the SF-MPQ), supporting the observations of other investigators.^17,26^ Upper limb disability was also reduced after GMI treatment, which is also in accordance with previous reports.^27^ Despite the reduced disability in the affected limb, no clinically significant change in grip strength was observed. These results are in opposition with those of Lagueux et al., who showed an increased in grip force but no change in the perceived function of the affected limb, suggesting that the effects of GMI may be variable.^26^

### Limitations

This study is a single-subject case report performed in the absence of a neuronavigation system. Our results should be interpreted with caution, because they provide evidence that undoubtedly will need to be tested in larger-scale studies. Neuronavigation systems are designed to help position TMS coils over the optimal stimulation site, maintaining a constant position/orientation of the coil throughout testing. This is particularly important, because these factors can have a profound impact on MEP amplitude.^28,29^ Yet, it must be noted that the added value of neuronavigation is still argued, because some research teams have found no differences in MEP amplitude variability and reproducibility between navigated and nonnavigated TMS protocols.^30^ In the present case report, the position and orientation of the coil were carefully monitored by using landmarks traced on the patient’s swim cap. During all of the testing sessions, the experimenter frequently reassessed the position of the coil to ensure that it remained over the stimulation site, maintaining the correct orientation. This method is part of best practices in TMS.^31,32^

An additional limitation concerns the method used to evaluate the strength of the corticospinal projections. For our patient, the strength of the corticospinal projections was assessed using a shortened procedure, based on the evaluation of MEP amplitude obtained at 110% and 130% of rMT. Traditionally, the strength of the corticospinal projections is assessed more thoroughly by obtaining input–output recruitment curves.^20,33^Input–output recruitment curves depict the rise of MEP size with increasing TMS intensities (e.g., 90%, 110%, 130%, and 150% of rMT) and are more suitable to evaluate corticomotor changes over time.^20,33,34^ Because of the time constraints imposed by the clinical environment, a shorter procedure was required, and a metric inspired by Lefaucheur and colleagues, based on the calculation of a delta score between two TMS intensities, was used.^21^ Future studies looking into the corticomotor changes of CRPS patients using complete input–output recruitment curves are warranted.

Finally, the presence of edema is another important issue that must be considered, because this factor can substantially influence EMG recordings and MEP amplitude measures. Although the patient showed slight edema during the initial visit, no significant changes were noted throughout the study. In addition, it should be noted that edema will affect the MEP responses obtained at both 110% and 130% of the rMT (therefore probably having only a limited impact on the delta score). Thus, it is unlikely that the changes in the strength of the corticospinal projections noted between the initial and final visits in our patient could be attributable to fluctuations in edema.

### Conclusion

To conclude, the present case report suggests that CNS reorganizations can be observed in the motor system of a patient with CRPS. The reduction in pain and disability following effective rehabilitation treatments (in this case, GMI) appears to be linked to a reduction of motor system changes. Future studies are needed to confirm these findings and to better understand the interaction between the motor system and pain in patients with CRPS.
